# NPC86 Increases LncRNA Gas5 In Vivo to Improve Insulin Sensitivity and Metabolic Function in Diet-Induced Obese Diabetic Mouse Model

**DOI:** 10.3390/ijms26083695

**Published:** 2025-04-14

**Authors:** Anna Kharitonova, Rekha S. Patel, Brenna Osborne, Meredith Krause-Hauch, Ashley Lui, Gitanjali Vidyarthi, Sihao Li, Jianfeng Cai, Niketa A. Patel

**Affiliations:** 1 James A. Haley Veteran’s Hospital, Research Service, 13000 Bruce B Downs Blvd, Tampa, FL 33612, USA; kharitonova@usf.edu (A.K.); rekha.patel1@va.gov (R.S.P.); brenna.osborne@va.gov (B.O.); meredith.krause-hauch@va.gov (M.K.-H.); gitanjali.vidyarthi@va.gov (G.V.); 2 Department of Chemistry, University of South Florida, Tampa, FL 33620, USA; sihaoli@usf.edu (S.L.); jianfengcai@usf.edu (J.C.); 3 Department of Molecular Oncology, Moffitt Cancer Center, Tampa, FL 33612, USA; ashley.lui@moffitt.org; 4 Department of Molecular Medicine, University of South Florida, Tampa, FL 33612, USA

**Keywords:** type 2 diabetes mellitus (T2D), long noncoding RNA (lncRNA), gas5, insulin resistance, glucose metabolism, NPC86, diet-induced obese diabetic (DIOD), RNA sequencing (RNAseq), metabolic pathways, inflammation and insulin signaling

## Abstract

In the United States, an estimated 38 million individuals (10% of the population) have type 2 diabetes mellitus (T2D), while approximately 97.6 million adults (38%) have prediabetes. Long noncoding RNAs (lncRNAs) are critical regulators of gene expression and metabolism. We were the first to demonstrate that lncRNA Growth Arrest-Specific Transcript 5 (GAS5 (human)/gas5 (mouse)) is decreased in the serum of T2D patients and established GAS5 as a biomarker for T2D diagnosis and onset prediction, now validated by other groups. We further demonstrated that GAS5 depletion impaired glucose uptake, decreased insulin receptor levels, and inhibited insulin signaling in human adipocytes, highlighting its potential as a therapeutic target in T2D. To address this, we developed NPC86, a small-molecule compound that stabilizes GAS5 by disrupting its interaction with UPF-1, an RNA helicase involved in nonsense-mediated decay (NMD) that regulates RNA stability. NPC86 increased GAS5 and insulin receptor (IR) levels, enhanced insulin signaling, and improved glucose uptake in vitro. In this study, we tested the efficacy of NPC86 in vivo in a diet-induced obese diabetic (DIOD) mouse model, and NPC86 treatment elevated gas5 levels, improved glucose tolerance, and enhanced insulin sensitivity, with no observed toxicity or weight changes. A transcriptomics analysis of adipose tissue revealed the upregulation of insulin signaling and metabolic pathways, including oxidative phosphorylation and glycolysis, while inflammatory pathways were downregulated. These findings highlight NPC86’s therapeutic potential in T2D.

## 1. Introduction

Diabetes is a chronic and progressive metabolic disorder characterized by hyperglycemia due to impaired insulin secretion, insulin action, or both [1]. Type 2 diabetes mellitus (T2D) accounts for approximately 90–95% of all adult diabetes cases globally and remains a significant public health issue [2]. In the United States, an estimated 38 million individuals, or about 10% of the population, are living with T2D, while approximately 38% of adults—totaling 97.6 million people—have prediabetes [3]. Additionally, it was projected that prediabetes will continue to rise among the population, potentially reaching 107.7 million individuals by 2030 [4].

The prevalence of diabetes is associated with a broad spectrum of complications, including cardiovascular disease, nephropathy, neuropathy, retinopathy, and impaired wound healing, which contribute to high morbidity and mortality rates [5]. The burden of T2D is even more pronounced among veterans, with one in four veterans with diabetes, compared to the 1 in 10 rate seen in civilian populations [6]. Additionally, a staggering 78% of veterans are classified as obese, a major risk factor for diabetes progression [7,8]. The increased incidence of T2D in the veteran population can be attributed to a combination of service-related risk factors, including chronic injuries, psychological stressors, and hazardous environmental exposures that are unique to military service [9]. Given the increasing prevalence of T2D and its complications, there is an urgent need for novel therapeutic strategies that go beyond symptom management [10]. Current treatments primarily focus on glycemic control and reducing the risk of complications [11]. Despite significant advancements in diabetes care, no curative treatment exists, and many patients experience progressive disease worsening over time [12,13]. Therefore, identifying molecular targets that can modulate early metabolic changes of T2D pathophysiology remains a critical area of research. Long noncoding RNAs (lncRNAs) are multifunctional molecular regulators of cellular processes, and their aberrant expression is shown to be associated with disease states.

LncRNAs represent a diverse class of RNA molecules, exceeding 200 nucleotides in length, that are not translated to protein. LncRNAs are key regulators of gene expression, cellular physiology, and pathogenesis [10,11,12,13,14,15]. Growth Arrest-Specific 5 (GAS5 (human)/gas5 (mouse)) is a lncRNA with multiple biological functions, including regulation of cell proliferation and survival, as well as its potential involvement in disease pathogenesis [16]. The GAS5 gene also harbors encoding sequences for an array of small nucleolar RNAs (snoRNAs) [17]. Despite the generation of multiple GAS5 transcript isoforms via alternative splicing, exon 12—the final exon—remains consistently preserved and displays a high level of conservation across mouse and human genomes [18]. None of the GAS5 splice variants undergo translation into protein due to a premature termination codon (PTC), rendering gas5 susceptible to nonsense-mediated RNA decay (NMD) [19]. UPF-1, a pivotal helicase responsible for NMD, binds to the 3′ region of gas5, initiating the NMD process. The regulation of GAS5 levels is governed by its degradation process [20].

We were the first research group to demonstrate that GAS5 expression was significantly reduced in both the serum of T2D patients [21] and diabetic adipocytes [22]. Additionally, we identified GAS5 as a key regulator of insulin signaling and insulin receptor expression in human adipocytes, highlighting its potential as a therapeutic target [22]. Subsequent studies have independently confirmed our findings, consistently reporting decreased GAS5 levels in T2D, reinforcing its potential role in T2D pathogenesis [23,24,25,26]. To counteract GAS5 downregulation, we developed NPC86, a small-molecule compound that stabilizes GAS5 by preventing its degradation by UPF1 via the nonsense-mediated pathway (NMD). NPC86 disrupts the interaction between GAS5 and UPF-1, thereby stabilizing GAS5 levels. In diabetic adipocytes, NPC86 increases GAS5 levels, enhancing insulin receptor expression and insulin signaling [22].

To assess the in vivo efficacy of NPC86 in this study, we evaluated the systemic effects of NPC86 in a diet-induced obese diabetic (DIOD) C57BL/6J mouse model. We evaluated the target engagement, safety, and efficacy of NPC86. We undertook a transcriptomics analysis to identify the genes and pathways affected by NPC86 treatments. Our results establish NPC86 as a promising therapeutic strategy to enhance gas5 expression, improve insulin sensitivity, and mitigate metabolic dysfunction in T2D.

## 2. Results

### 2.1. DIOD Mice Have Impaired Glucose Tolerance and Low Gas5 Expression Across Multiple Tissues

**Figure 1:**
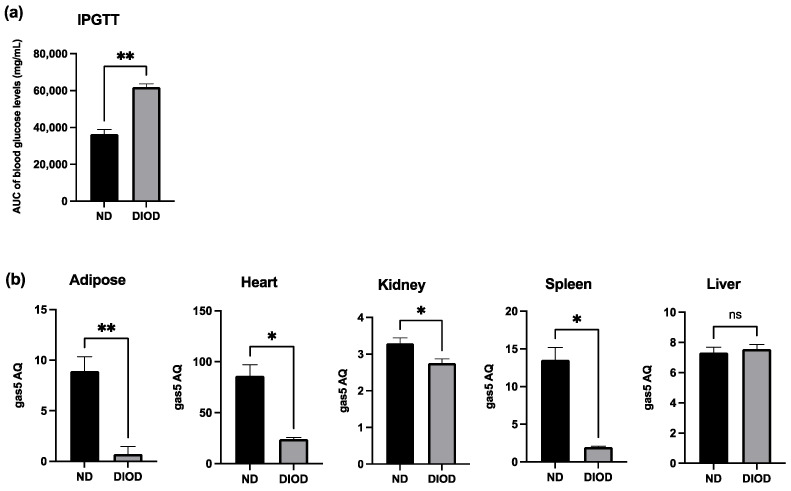
DIOD impairs glucose tolerance and downregulates gas5 expression in multiple tissues. (**a**) Intraperitoneal Glucose Tolerance Test (IPGTT) in ND and DIOD C57BL/6J mice after an 8 h fast followed by intraperitoneal glucose injection (2 g/kg body weight). The area under the curve (AUC) was calculated to quantify glucose exposure over time using the trapezoidal rule, following the formula: AUC (mmol/L*min) = 1/2 × (BG 0 min + BG 30 min) × 30 min + 1/2 × (BG 30 min + BG 60 min) × 30 min + 1/2 × (BG 60 min + BG 90 min) × 30 + 1/2 × (BG 90 min + BG 120 min) × 30 min, where BG represents blood glucose values at each respective time point. A statistical analysis was performed in GraphPad Prism using an unpaired *t*-test. The data are presented as mean ± SEM; *n* = 3 per group; ** *p* < 0.01. (**b**) Total RNA was extracted from the adipose tissue of the ND and DIOD mice (*n* = 3 per group). Real-time qPCR was performed in triplicate using SYBR Green to measure the absolute quantification of gas5 expression and normalized to GAPDH. A statistical analysis was performed in GraphPad Prism using unpaired *t*-test. The data are presented as mean ± SEM; *n* = 3 per group; * *p* < 0.05, ** *p* < 0.01; ns = not significant.

To assess the impact of DIOD on systemic metabolic function, we performed an Intraperitoneal Glucose Tolerance Test (IPGTT) on lean non-diabetic (ND) and diet-induced obese diabetic (DIOD) C57BL/6J mice. Following a two-week acclimation period, the mice were fasted for 8 h before receiving an intraperitoneal injection of D-glucose solution (2 g/kg body weight). Blood samples were collected from the tail vein at 0, 30, 60, and 120 min post-injection to assess glucose clearance. The IPGTT analysis revealed (Figure 1a) that the DIOD mice exhibited significant impairments in glucose clearance compared to their ND counterparts, as quantified by the area under the curve (AUC) for blood glucose levels. AUC calculations confirmed a higher glucose exposure over time in the DIOD mice, indicating prolonged hyperglycemia and reduced glucose disposal.

Previously, our group demonstrated the downregulation of lncRNA GAS5 in human diabetic adipocytes, highlighting its potential role in metabolic dysregulation. To extend these findings, we evaluated gas5 expression in adipose tissue, heart, liver, spleen, and kidney samples from the ND and DIOD C57BL/6J mice using RT-qPCR. Our results (Figure 1b) demonstrated significant suppression of gas5 expression in the adipose, heart, kidney, and spleen tissues of the DIOD mice compared to their ND counterparts. However, no significant changes in gas5 expression were observed in the liver samples, suggesting that gas5 dysregulation in response to metabolic stress is tissue-specific in our cohort of DIOD mice. Our results also show varying levels of gas5 across organs, comparing ND to DIOD. The observed downregulation of gas5 across multiple tissues further implicates its potential role in the systemic metabolic alterations associated with diabetes.

### 2.2. NPC86 Treatment Increases Gas5 Expression in the Adipose, Cardiac, Renal, and Spleen Tissues of DIOD Mice

**Figure 2:**
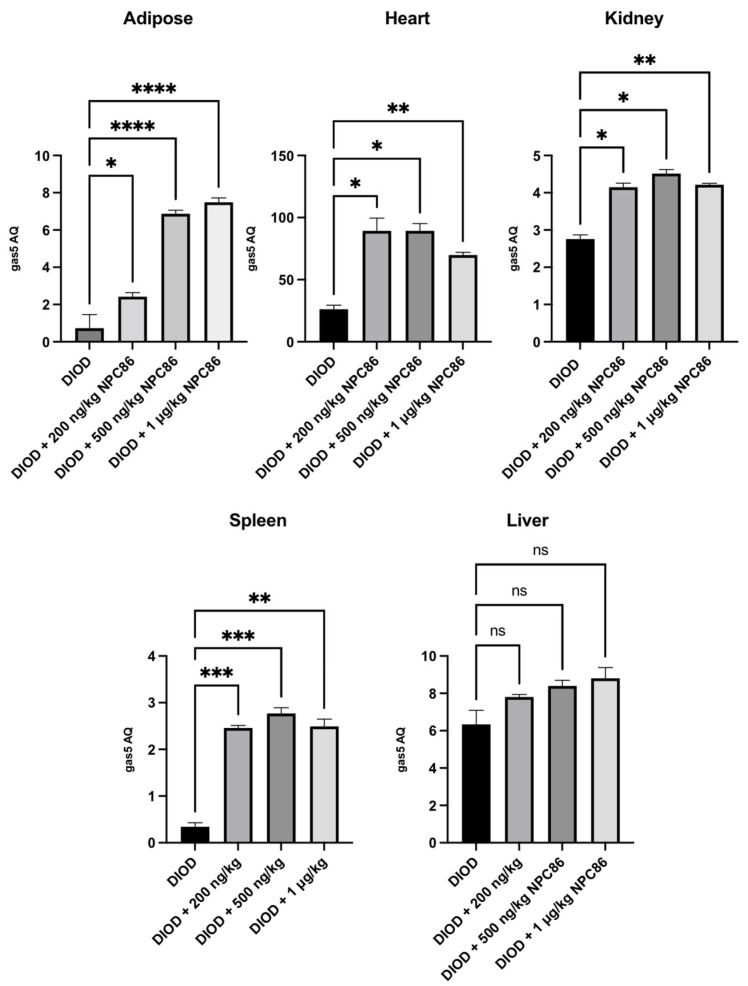
Total RNA was extracted from the kidney, spleen, heart, adipose, and liver tissues of DIOD and DIOD + NPC86 (200 ng/kg, 500 ng/kg, or 1 μg/kg) mice. Real-time qPCR was performed in triplicate using SYBR Green to measure the absolute quantification of gas5 expression and normalized to GAPDH. A statistical analysis was performed using a one-way ANOVA in GraphPad Prism. The data are presented as mean ± SEM; *n* = 3 per group; * *p* < 0.05, ** *p* < 0.01, *** *p* < 0.001 **** *p* < 0.0001; ns = not significant.

We have previously demonstrated the design and development of NPC86, a small-molecule compound that stabilizes GAS5 by inhibiting UPF1 binding to the GAS5 3′ region [22]. Given the observed suppression of gas5 expression in DIOD mice, we sought to determine whether NPC86 could upregulate its levels systemically. The DIOD mice were treated with intraperitoneal injections of NPC86 at doses of 200 ng/kg b.w., 500 ng/kg b.w., and 1 μg/kg b.w. daily for five consecutive days. An RT-qPCR analysis of RNA extracted from the adipose, heart, spleen, and kidney tissues revealed a tissue-specific response to NPC86 treatment (Figure 2). Notably, the liver did not show a response consistent with prior observations in the untreated DIOD mice. In the adipose tissue, the gas5 levels showed a modest increase at 200 ng/kg NPC86 but were significantly upregulated at 500 ng/kg b.w. and 1 μg/kg b.w., indicating a dose-dependent response. The highest expression was observed at 1 μg/kg b.w., suggesting that higher doses more effectively increased the gas5 levels in the DIOD mice. In kidney and spleen, the gas5 levels were markedly increased at all doses. In the heart, gas5 expression followed a dose-dependent trend, showing notable upregulation at 200 ng/kg b.w., which was further enhanced at 500 ng/kg b.w. At 1 μg/kg b.w., expression remained elevated but did not exceed the 500 ng/kg response. These findings confirm that NPC86 enhances gas5 expression in a tissue-specific manner, with spleen, kidney, heart, and adipose tissue showing significant responses. The data suggest that lower doses (200–500 ng/kg b.w.) are optimal for upregulating gas5 levels, while higher doses (1 μg/kg b.w.) do not provide additional regulatory effects.

### 2.3. Histopathological Analysis and Stable Body Weight Confirm NPC86 Safety

**Figure 3:**
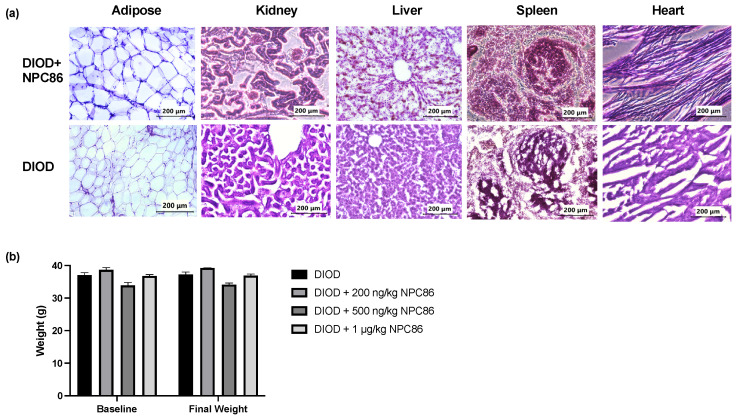
NPC86 treatment shows no signs of toxicity and does not alter body weight. (**a**) Representative hematoxylin and eosin (H&E)-stained sections of adipose, kidney, liver, spleen, and heart tissues from DIOD mice (bottom row) and NPC86-treated (500 ng/kg b.w.) DIOD mice (top row). (**b**) Body weight was measured at baseline and following a 5-day treatment regime with NPC86 treatment (200 ng/kg, 500 ng/kg, and 1 µg/kg) in DIOD mice. A statistical analysis was performed in GraphPad Prism using paired *t*-test. The data are presented as mean ± SEM; *n* = 3 per group.

To evaluate the safety of NPC86, we conducted a histological analysis using hematoxylin and eosin (H&E) staining of liver, spleen, kidney, heart, and adipose tissues from DIOD mice and DIOD mice treated with NPC86. Across all examined tissues, the histological analysis (Figure 3a) demonstrated preserved architecture, with no evidence of toxicity. The liver and spleen sections displayed normal histological features without any signs of structural disruptions. An examination of the kidney tissues revealed no pathological changes. The cardiac tissue maintained its characteristic myocardial architecture, and the adipose tissue displayed normal adipocyte morphology. In addition to the histopathological analysis, body weight measurements remained stable across all treatment groups throughout the duration of the study (Figure 3b). Collectively, these findings provide strong evidence supporting the safety of NPC86, reinforcing its potential for further therapeutic development.

### 2.4. NPC86 Improves Glucose Tolerance in DIOD Mice

**Figure 4:**
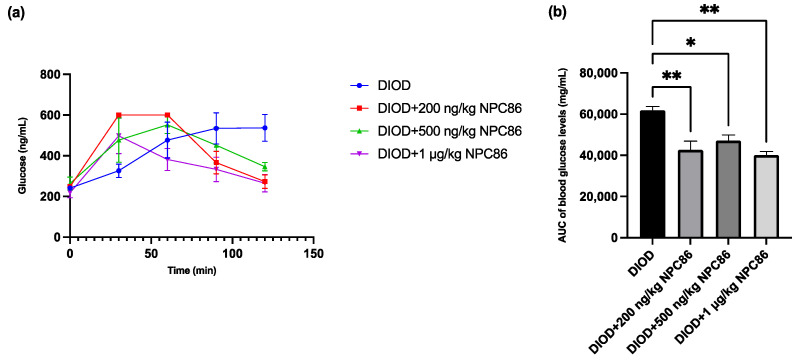
NPC86 improves glucose clearance in DIOD mice. (**a**) Blood glucose levels were measured during an intraperitoneal glucose tolerance test (IPGTT) following an 8 h fasting period. The mice received an intraperitoneal injection of D-glucose (2 g/kg body weight), and blood glucose levels were recorded at 0, 30, 60, 90, and 120 min post-injection. Data are presented as mean ± SEM (*n* = 3 per group). (**b**) The area under the curve (AUC) was calculated to quantify glucose exposure over time using the trapezoidal rule, following the formula: AUC (mmol/L*min) = 1/2 × (BG 0 min + BG 30 min) × 30 min + 1/2 × (BG 30 min + BG 60 min) × 30 min + 1/2 × (BG 60 min + BG 90 min) × 30 + 1/2 × (BG 90 min + BG 120 min) × 30 min, where BG represents blood glucose values at each respective time point. Statistical comparisons between groups were performed using one-way ANOVA, *n* = 3 per group. Statistical significance is indicated as * *p* < 0.05 and ** *p* < 0.01.

After completing a 5-day NPC86 treatment regimen, we reassessed glucose metabolism by performing IPGTT across the DIOD and NPC86-treated groups to determine the effect of NPC86 on systemic glucose regulation. Prior to treatment, the DIOD mice exhibited significant glucose intolerance, as indicated by their delayed return to baseline glucose levels following glucose administration. Using the same IPGTT protocol, a post-treatment analysis demonstrated that NPC86 significantly improved glucose clearance in the DIOD mice. Compared to their pre-treatment profiles, the NPC86-treated DIOD mice exhibited a response trajectory that resembled that of the ND mice, indicating that NPC86 enhances glucose metabolism and may ameliorate insulin resistance in diet-induced obesity (Figure 4).

### 2.5. NPC86 Improves Metabolic Function in DIOD Mice: Insights from an RNAseq Analysis

To understand the transcriptomic impact of NPC86, we isolated RNA from the adipose tissue of ND mice, DIOD mice, and DIOD mice treated with NPC86 (500 ng/kg b.w. and 1 μg/kg b.w.). Isolated RNA samples were processed using next-generation RNA sequencing (RNAseq), which provided significant insights into the differentially expressed genes and pathways affected in DIOD and DIOD + NPC86 treatment. The results show substantial fold enrichment in several pathways critical to metabolism and disease pathogenesis, such as insulin signaling (3.5-fold), AMPK (4.6-fold), oxidative phosphorylation (7.80-fold), cholesterol metabolism (4.75-fold), pyruvate metabolism (5.29-fold), and overall metabolic pathways (2.45-fold). Carbon metabolism (5.2-fold) was also enriched, along with glycolysis/gluconeogenesis (3-fold), fat digestion and absorption (3.4-fold), cGMP-PKG signaling (2.1-fold), and fatty acid biosynthesis (3.8-fold), indicating a broad spectrum of metabolic processes influenced by NPC86 treatment in DIOD mice.

**Figure 5:**
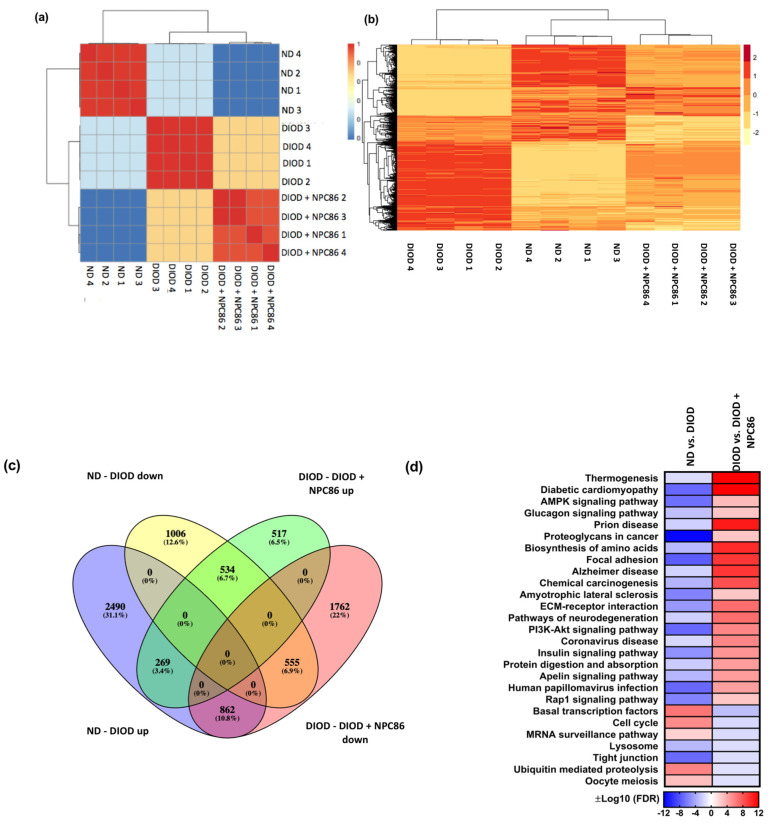
Distance heatmap, hierarchical clustering, differential gene expression, and pathway enrichment analysis of RNAseq data. (**a**) Distance heatmap showing the correlation of samples within their respective groups. The color scale ranges from blue (low correlation) to red (high correlation), with hierarchical clustering applied to group samples with similar transcriptomic signatures. (**b**) Hierarchical clustering heatmap of differentially expressed genes across experimental conditions. Rows represent genes, and columns represent individual samples. The expression values are Z-score-normalized, with red indicating upregulation and yellow indicating downregulation. The clustering demonstrates that the NPC86-treated (500 ng/kg b.w. and 1 μg/kg b.w.) DIOD mice exhibit a gene expression profile distinct from that of the untreated DIOD mice and more closely aligned with that of the ND controls, suggesting that NPC86 treatment reprograms the transcriptome toward a metabolically healthier state. (**c**) Venn diagram illustrating the overlap of differentially expressed genes (DEGs) among the experimental groups. The analysis shows that 1006 genes were downregulated in the DIOD mice compared to in the ND controls, while NPC86 treatment (500 ng/kg b.w. and 1 μg/kg b.w.) upregulated 517 genes, indicating a partial reversal of DIOD-associated transcriptional changes. Additionally, 555 genes that were upregulated in the DIOD mice compared to the ND controls were significantly downregulated following NPC86 treatment. A total of 534 genes overlapped between the groups, representing core transcriptional changes induced by NPC86 to promote metabolic balance. (**d**) Pathway enrichment heatmap displaying differentially regulated metabolic, inflammatory, and signaling pathways across experimental groups. The color scale represents log-transformed false discovery rate (FDR) −adjusted *p*-values, indicating pathway significance. Key metabolic pathways such as insulin signaling, AMPK signaling, and the biosynthesis of amino acids were enriched, suggesting that NPC86 plays a role in restoring metabolic homeostasis in DIOD mice.

To visualize these transcriptomic changes, multiple analytical approaches were applied. A confusion matrix (Figure 5a) and hierarchical clustering heatmap (Figure 5b) demonstrated that the NPC86-treated DIOD mice exhibited an expression profile distinct from that of the untreated DIOD mice and more closely aligned with that of the ND controls. This finding suggests that NPC86 may effectively reprogram the transcriptome toward a metabolically healthier state.

The differential gene expression analysis, represented by a Venn plot (Figure 5c), revealed a substantial overlap between genes regulated in response to NPC86 treatment. In the DIOD mice, 1006 genes were downregulated compared to in the ND controls, while NPC86 treatment upregulated 517 genes, demonstrating a partial reversal of DIOD-induced dysregulation. Furthermore, 555 genes that were upregulated in the DIOD mice compared to that in the ND controls were significantly downregulated following NPC86 administration. Notably, 534 genes overlapped across groups, highlighting core transcriptional changes induced by NPC86 that enhance metabolic balance.

A pathway enrichment heatmap (Figure 5d) further demonstrated that key metabolic, inflammatory, and signaling pathways were differentially regulated across experimental groups. Several pathways critical to metabolism and disease pathogenesis were enriched, supporting the role of NPC86 in regulating metabolic homeostasis.

**Figure 6:**
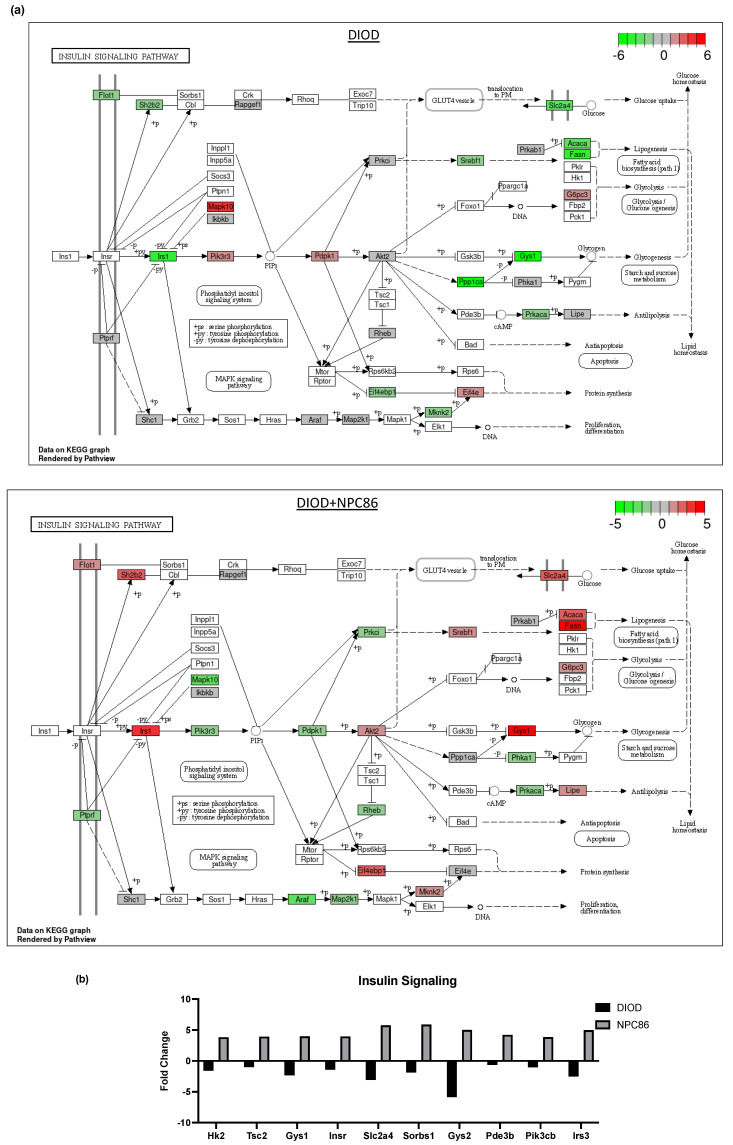

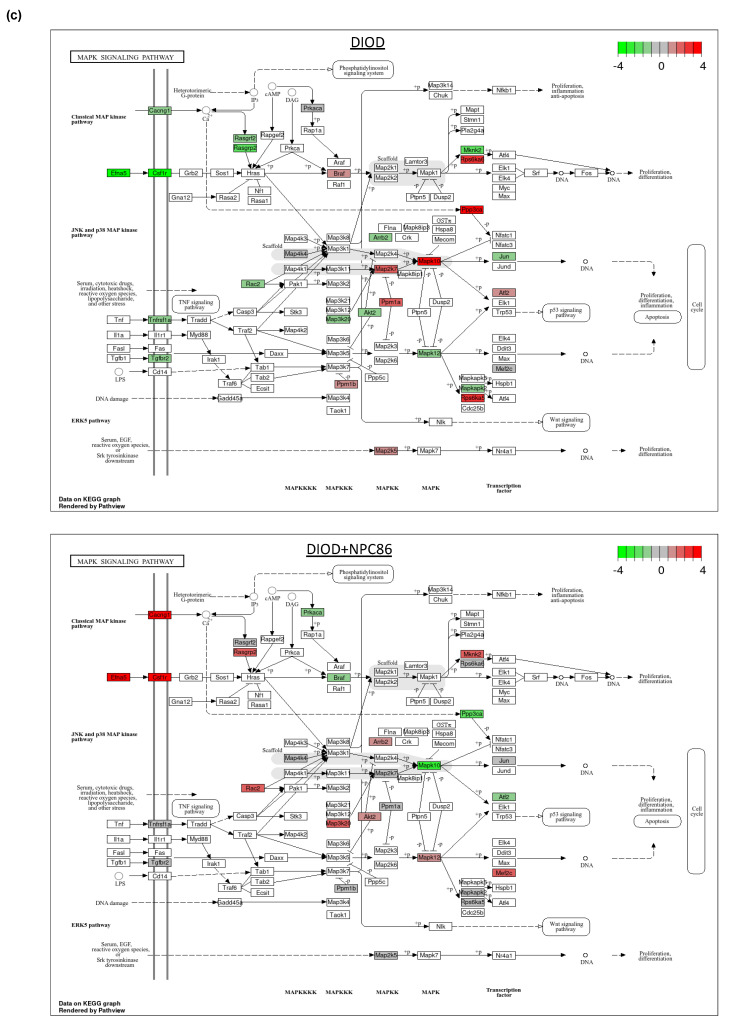

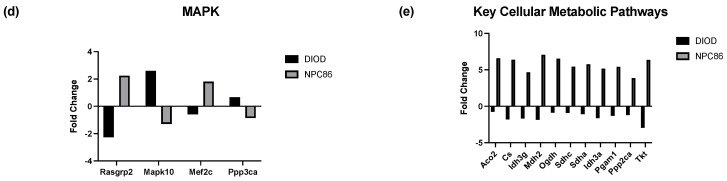
Impact of NPC86 on insulin signaling and inflammatory pathways in adipose tissue. (**a**) KEGG pathway diagrams illustrating changes in the insulin signaling pathway in the DIOD (top) and DIOD + NPC86 (500 ng/kg b.w. and 1 μg/kg b.w.; bottom) groups. The color gradient represents log2 fold changes in gene expression, with red indicating upregulation and green indicating downregulation. (**b**) Bar plot illustrating fold-change values of differentially expressed genes (DEGs) involved in insulin signaling (DIOD vs. DIOD + NPC86 (500 ng/kg b.w. and 1 μg/kg b.w.) mice). The *x*-axis represents the log2 fold-change in gene expression between the DIOD and NPC86-treated mice. Positive values indicate genes that are upregulated following NPC86 treatment, whereas negative values represent genes that are downregulated. (**c**) KEGG pathway analysis of the MAPK signaling pathway in DIOD (left) and NPC86-treated (500 ng/kg b.w. and 1 μg/kg b.w.; right) mice. Colored boxes indicate log2 fold-change values, with red representing upregulation and green representing downregulation of gene expression. (**d**) Bar plot showing fold-change values of genes involved in the MAPK signaling pathway between DIOD and DIOD + NPC86 mice (500 ng/kg b.w. and 1 μg/kg b.w.). Positive values indicate upregulation, while negative values indicate downregulation following NPC86 treatment, highlighting its regulatory effects on MAPK-related gene expression. (**e**) Bar plot showing the number of upregulated and downregulated genes involved in key cellular metabolic pathways in DIOD vs. DIOD + NPC86-treated mice (500 ng/kg b.w. and 1 μg/kg b.w.). The *x*-axis represents the number of differentially expressed genes (DEGs), while the bars indicate whether genes were upregulated or downregulated following NPC86 treatment.

These changes aligned with the improvements in insulin sensitivity and metabolic homeostasis. NPC86 treatment reversed the downregulation of insulin signaling (Figure 6a), PI3K-Akt signaling, and AMPK pathways observed in DIOD mice, modulating pathways critical to glucose homeostasis, lipid metabolism, and mitochondrial function. Additionally, genes involved in cellular stress responses and immune system regulation were significantly modulated, suggesting that NPC86 plays a role in both metabolic and inflammatory reprogramming.

Key insulin signaling genes (Figure 6b), such as *INSR*, *IRS3*, *Pik3cb*, and *Sorbs1*, were significantly upregulated following NPC86 treatment, showing fold changes consistent with improved insulin sensitivity. *Gys2* and *Pde3b*, which were downregulated in DIOD mice, were upregulated following NPC86 treatment, indicating enhanced glycogen synthesis and lipid signaling regulation [27,28]. Notably, *Slc2a4* (*GLUT4*), a critical glucose transporter, showed a significant increase in expression in the NPC86-treated mice compared to that in the DIOD controls, suggesting enhanced glucose uptake capacity in adipose tissue [29]. NPC86 treatment significantly modulated the MAPK pathway, a key regulator of metabolic stress responses (Figure 6c,d). Notably, Rasgrp2 expression was upregulated, suggesting a potential role in enhancing cellular adaptation to metabolic stress [30].

Within the framework of affected pathways, we identified several genes in glycolysis and the TCA cycle that were dysregulated in DIOD mice and modulated by NPC86 (Figure 6e). Genes involved in glycolysis and the TCA cycle that were dysregulated in DIOD mice, such as *Pgam1*, *Cs*, *Idh3g*, *Mdh2*, *Ogdh*, *Sdha*, and *Idh3a*, were significantly upregulated following NPC86 treatment, suggesting enhanced glycolytic flux and mitochondrial energy production. Genes involved in lipid metabolism, such as *Lpl* and *Abca1*, were significantly changed in DIOD vs. DIOD + NPC86 mice. Both genes, which were downregulated in DIOD mice, exhibited enhanced expression levels in NPC86-treated mice, suggesting enhanced lipid metabolism and cholesterol efflux, respectively [31,32]. Additionally, *Acox1*, involved in peroxisomal beta-oxidation, and *Pparg*, a key regulator of adipogenesis and insulin sensitivity, showed increased expression with NPC86 treatment, highlighting improvements in lipid signaling and fatty acid metabolism [33,34]. Genes involved in oxidative stress response, including *Txn1*, *Prdx1*, and *Gpx1*, were also significantly upregulated with NPC86 treatment. These genes play key roles in counteracting oxidative damage and maintaining cellular homeostasis, suggesting that NPC86 may contribute to improved oxidative stress resilience in adipose tissue [35,36,37,38].

The observed changes extended to pathways that regulate metabolic flexibility and stress responses. *Tsc2*, a key player in the AMPK and mTOR signaling pathways, displayed increased expression patterns, further implicating NPC86 in enhancing metabolic resilience [39,40]. Additionally, genes such as *Aco2* and *Sdhc*, which regulate oxidative phosphorylation and mitochondrial function, were significantly modulated by NPC86, indicating improved mitochondrial efficiency and reduced oxidative stress [41,42].

**Figure 7:**
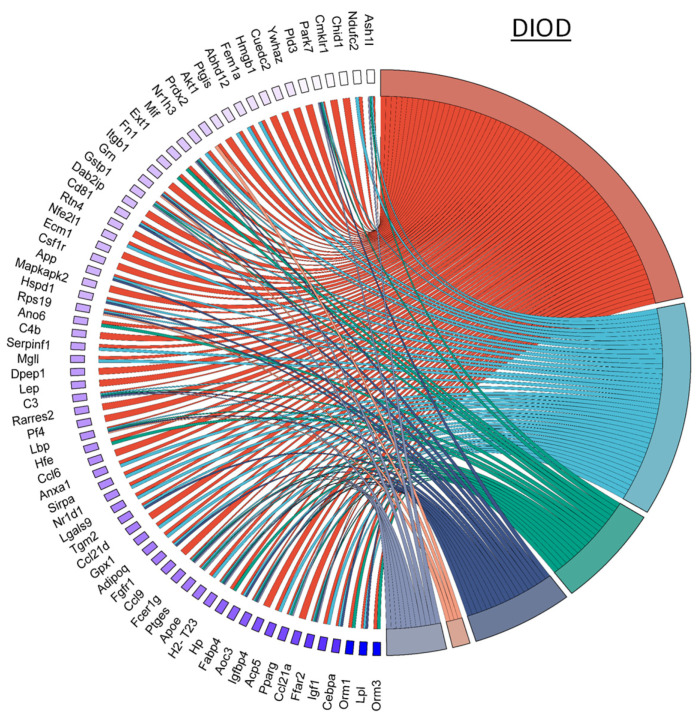

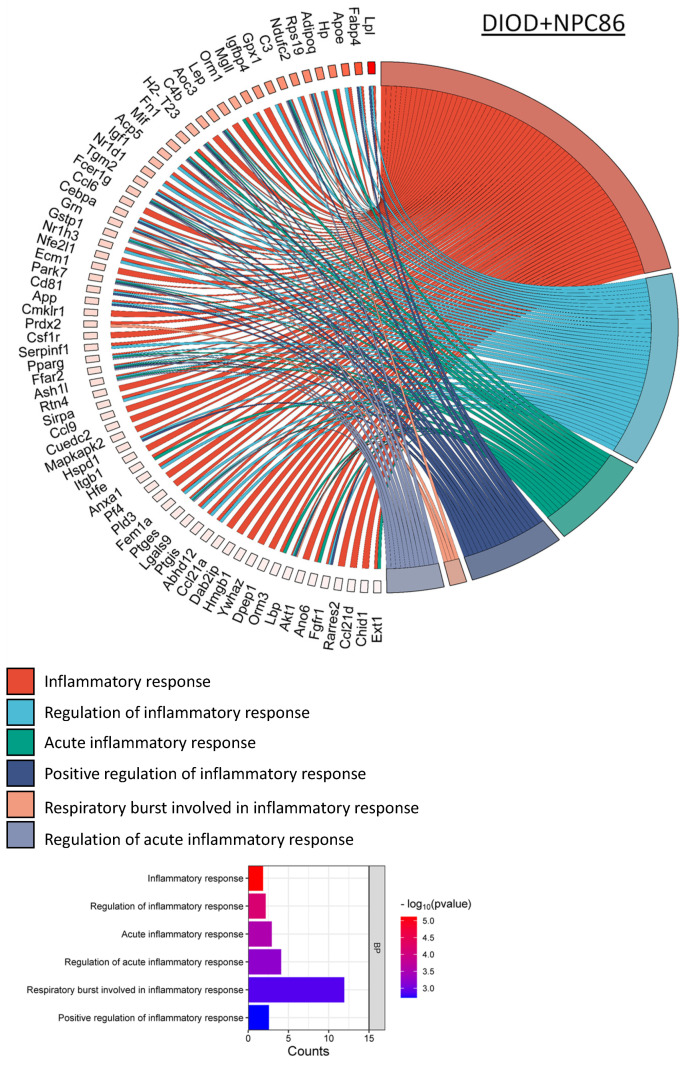
NPC86 modulates inflammatory gene networks in adipose tissue. Chord diagrams depict gene-pathway interactions related to inflammatory responses in DIOD (left) and NPC86-treated (right) mice. Each arc represents a gene associated with a specific inflammatory pathway, color-coded according to its pathway classification. Compared to the DIOD mice, the NPC86-treated mice exhibit a pronounced downregulation of genes involved in acute inflammation, respiratory burst, and immune activation. The pathway enrichment analysis highlights the significant repression of key inflammatory pathways, including cytokine signaling and stress-response pathways, in the NPC86-treated mice. The bar plot illustrates the enrichment of inflammatory response-related pathways. The *x*-axis represents the number of DEGs associated with each pathway, while the color gradient represents the statistical significance (−log10(*p*-value)). NPC86 treatment significantly downregulated pathways related to inflammation, including acute inflammatory response and positive regulation of inflammatory response, supporting its role in mitigating inflammation-associated dysregulation.

NPC86 also had a pronounced effect on inflammatory gene networks, as illustrated by chord diagrams depicting gene–pathway interactions related to inflammation (Figure 7). Compared to the untreated DIOD mice, the NPC86-treated mice exhibited a significant downregulation of genes involved in acute inflammation, respiratory burst, and immune activation. A pathway enrichment analysis confirmed that key inflammatory pathways, including cytokine signaling and stress-response pathways, were significantly repressed following NPC86 administration, suggesting that NPC86 exerts an anti-inflammatory effect in adipose tissue. Notably, NPC86 treatment significantly downregulated key inflammatory genes in adipose tissue, particularly those involved in acute inflammation, cytokine signaling, and immune activation.

### 2.6. NPC86 Enhances Insulin Receptor Expression in DIOD Mice

**Figure 8:**
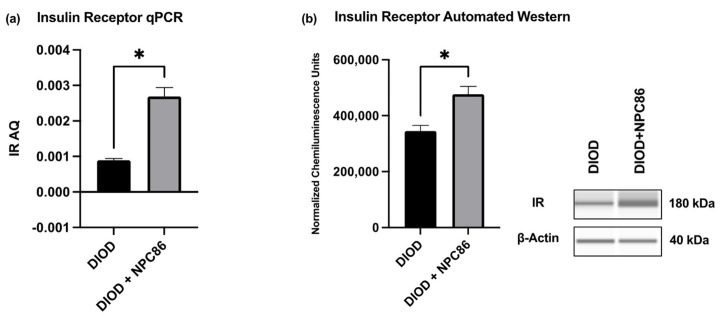
NPC86 enhances insulin receptor expression in DIOD mice. (**a**) Total RNA was extracted from the adipose tissue of DIOD and DIOD + NPC86 mice (200 ng/kg b.w.). Real-time qPCR was performed in triplicate using SYBR Green to measure the absolute quantification of IR expression and normalized to GAPDH. A statistical analysis was performed in GraphPad Prism using an unpaired *t*-test (*n* = 3 per group). The data are presented as mean ± SEM; * *p* < 0.05. (**b**) An automated Western blot analysis using JESS was performed on adipose tissue from DIOD and DIOD + NPC86 (200 ng/kg b.w.) mice using antibodies against IR and β-actin. Representative blots and the quantification of chemiluminescence units are shown. A statistical analysis was performed in GraphPad Prism using an unpaired *t*-test (*n* = 3 per group). The data are presented as mean ± SEM, with statistical significance indicated as * *p* < 0.05.

To validate the transcriptomic findings and assess the impact of NPC86 on insulin signaling, we quantified the IR mRNA and protein levels in adipose tissue. An RT-qPCR analysis of total RNA extracted from the adipose tissue of DIOD and NPC86-treated DIOD mice (200 ng/kg b.w.) revealed a significant increase in IR mRNA levels following NPC86 administration (Figure 8a). These transcript-level findings were further supported at the protein level by an automated Western blot analysis using the ProteinSimple JESS system. Western blot quantification confirmed a marked elevation in IR protein expression in the NPC86-treated mice (200 ng/kg b.w.) compared to that in the untreated DIOD controls (Figure 8b), consistent with the RNAseq data demonstrating an upregulation of INSR, the gene encoding the insulin receptor.

### 2.7. NPC86 Enhances AKT Phosphorylation in DIOD Mice

**Figure 9:**
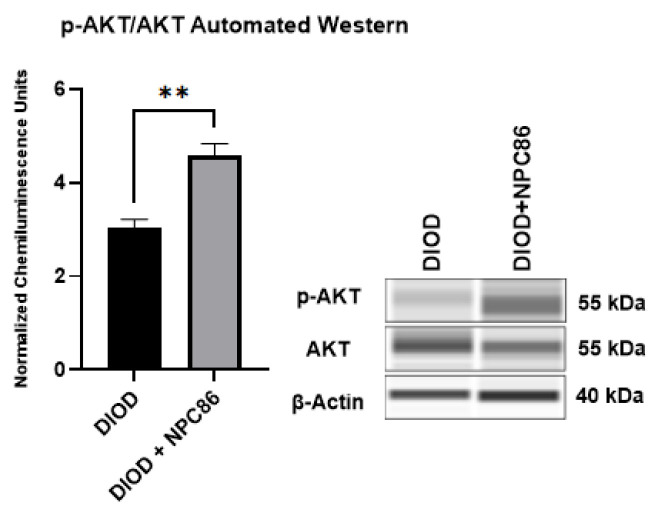
NPC86 treatment increases pAKT/AKT in DIOD mice. An automated Western blot analysis using JESS was performed on adipose tissue from DIOD and DIOD + NPC86 mice using antibodies against p-AKT (Ser473), total AKT (1/2/3), and β-actin. The phosphorylated AKT to total AKT (p-AKT/AKT) ratio is significantly elevated following NPC86 treatment (1 μg/kg b.w.). Representative blots and quantification are provided. A statistical analysis was performed in GraphPad Prism using an unpaired *t*-test (*n* = 3 per group). The data are presented as mean ± SEM, with statistical significance indicated as ** *p* < 0.01.

Since NPC86 treatment increased IR, we evaluated the impact of NPC86 treatment on insulin signaling. We conducted an automated Western blot analysis using the ProteinSimple JESS system to quantify phosphorylated AKT at Ser473 (p-AKT) and total AKT (1/2/3) levels in adipose tissue. Since AKT phosphorylation is a critical step in insulin signaling, increased p-AKT levels indicate enhanced activation of this pathway, which is essential for glucose uptake and metabolic regulation. Our results (Figure 9) demonstrated a significant increase in p-AKT levels in the NPC86-treated DIOD mice compared to that in the untreated DIOD controls. These findings align with our RNAseq results, which showed upregulation of key insulin signaling components in response to NPC86 treatment. These results demonstrate that NPC86 enhances insulin sensitivity by stabilizing gas5 levels.

### 2.8. NPC86 Reduces Inflammatory Cytokines in DIOD Mice

**Figure 10:**
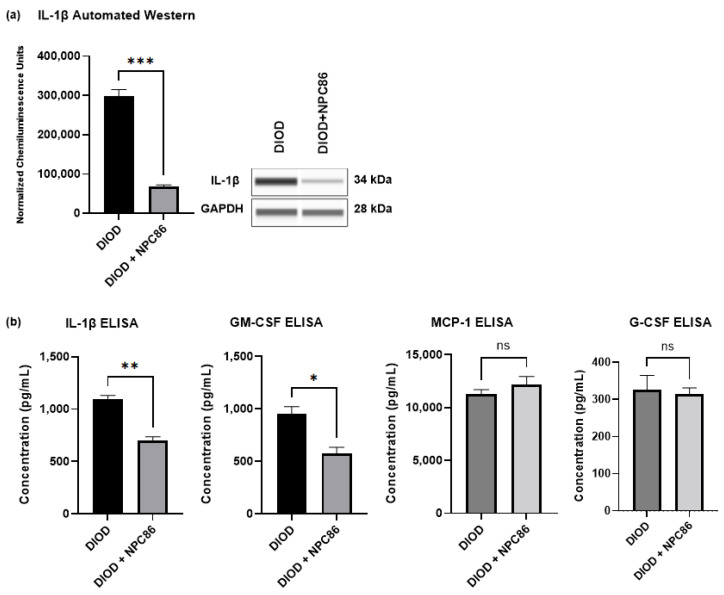
NPC86 reduces IL-1β protein levels. (**a**) An automated Western blot analysis using JESS was performed on adipose tissue from DIOD and DIOD + NPC86 (500 ng/kg b.w.) mice using antibodies against IL-1β and GAPDH. Representative blots and quantification are displayed. A statistical analysis was performed in GraphPad Prism using an unpaired *t*-test (*n* = 3 per group). The data are presented as mean ± SEM, with statistical significance indicated as *** *p* < 0.001. (**b**) The serum concentrations of IL-1β, GM-CSF, G-CSF, and MCP-1 were measured by ELISA in DIOD and DIOD + NPC86 (500 ng/kg and 1 μg/kg b.w.) mice. A statistical analysis was performed in GraphPad Prism using an unpaired *t*-test (*n* = 3 per group). The data are presented as mean ± SEM, with statistical significance indicated as * *p* < 0.05, ** *p* < 0.01.

The RNAseq analysis indicated that NPC86 treatment downregulated pro-inflammatory pathways. To determine the impact of NPC86 on inflammatory signaling, we first quantified the IL-1β protein levels in adipose tissue using automated Western blotting in ProteinSimple JESS. IL-1β expression was significantly lower in the NPC86-treated DIOD mice (500 ng/kg b.w.) compared to that in the untreated DIOD mice (*p* < 0.001), indicating a strong anti-inflammatory effect (Figure 10a).

Then, to assess whether NPC86 also exerted systemic anti-inflammatory effects, we performed an ELISA-based quantification of circulating cytokines in the serum of DIOD and DIOD + NPC86 (500 ng/kg and 1 μg/kg b.w.) mice, including IL-1β, GM-CSF, G-CSF, and MCP-1 (Figure 10b). Compared to untreated DIOD mice, NPC86 significantly reduced the serum levels of IL-1β and GM-CSF. IL-1β plays a critical role in coordinating both immune and metabolic functions by influencing insulin production. Additionally, the induction of β-cell death by IL-1β, is known to contribute to the onset and progression of type 2 diabetes [43,44]. The serum levels of GM-CSF are known to be elevated in individuals with T2D and correlate positively with glycated hemoglobin, underscoring its association with glycemic dysregulation [45]. However, the MCP-1 and G-CSF levels did not show significant changes following NPC86 treatment.

## 3. Discussion

The increasing prevalence of type 2 diabetes mellitus (T2D) presents a significant public health challenge, reinforcing the urgent need for therapeutic strategies that target the molecular drivers of insulin resistance. In the United States, T2D affects nearly 38 million individuals, with veterans experiencing more than four times the national incidence rate, placing them at disproportionately higher risk for metabolic complications.

In this context, long non-coding RNAs (lncRNAs) have gained recognition as key regulators of metabolic pathways. Among them, Growth-Arrest Specific Transcript 5 (GAS5), a member of the 5′-terminal oligopyrimidine (5′TOP) gene family, has emerged as a crucial modulator of cellular growth, immune response regulation, and apoptosis. GAS5 plays a key role in insulin signaling and metabolic regulation, and its downregulation has been implicated in metabolic dysregulation in diabetic models [46]. Previously, we demonstrated that GAS5 downregulation contributes to impaired insulin signaling and metabolic dysfunction in human adipocytes, positioning it as a potential therapeutic target.

We developed NPC86, a small-molecule compound designed to stabilize GAS5 expression by preventing its degradation, thereby enhancing its regulatory functions in insulin signaling and metabolic homeostasis. Previously, we demonstrated the binding properties, specificity, and kinetics of NPC86. We showed that NPC86 was highly specific to gas5 and did not affect other lncRNAs, such as MALAT1 and NEAT1, in adipocytes. NPC86 represents a pioneering RNA-targeting small molecule for metabolic diseases. In this study, we investigated the effects of NPC86 on GAS5 expression, insulin sensitivity, mitochondrial function, and inflammation in DIOD mice, providing novel insights into the therapeutic potential of lncRNA-targeting strategies.

Our study confirmed a systemic reduction in gas5 expression across multiple metabolic tissues in DIOD mice, including adipose, heart, kidney, and spleen, while liver gas5 levels remained unchanged. These findings suggest that lncRNA dysregulation contributes to obesity-associated metabolic dysfunction. NPC86 treatment significantly increased gas5 expression in adipose tissue, the heart, the kidneys, and the spleen, reinforcing its ability to stabilize gas5 in a tissue-specific manner. Hepatic gas5 levels did not vary between ND and DIOD, and no change was observed with NPC86 treatment.

Impaired insulin receptor (IR) signaling and AKT phosphorylation are hallmarks of insulin resistance [47,48]. The RNAseq analysis showed increased fold enrichment in key metabolic pathways, including insulin signaling, in response to NPC86 treatment. The RNAseq results were verified using qPCR and automated Western blots.

Glucose tolerance tests (IPGTT) further validated these findings, showing that NPC86-treated DIOD mice exhibited improved glucose clearance compared to untreated DIOD mice. These results suggest that NPC86 promotes insulin responsiveness by increasing gas5 levels and increasing insulin receptor activity in DIOD mice.

Beyond insulin signaling, NPC86 significantly influenced mitochondrial function and inflammatory responses in DIOD mice. The RNAseq analysis revealed the positive modulation of oxidative phosphorylation, glycolysis, and lipid metabolism pathways, suggesting that NPC86 enhances mitochondrial energy production and glucose utilization. Given that mitochondrial dysfunction is a hallmark of insulin resistance, these findings support the role of gas5 in mitochondrial regulation, in concurrence with a study demonstrating its mitochondrial localization and role in mitochondrial metabolic regulation [49].

Diabetes-related metabolic disorders are characterized by mitochondrial dysfunction and oxidative stress, leading to impaired ATP production and increased insulin resistance [50]. Our findings show that NPC86 upregulated genes involved in mitochondrial energy metabolism, including Pgam1, Cs, Idh3g, and Sdha, supporting the hypothesis that gas5 is involved in mitochondrial function regulation.

Additionally, NPC86 upregulated genes involved in oxidative stress regulation, including Txn1, Prdx1, and Gpx1, suggesting that NPC86 enhances antioxidant capacity and reduces oxidative stress. Since oxidative stress contributes to insulin resistance, these results indicate that NPC86 exerts its beneficial metabolic effects by improving both mitochondrial efficiency and cellular resilience to metabolic stress [51].

Inflammatory signaling is another key contributor to obesity-driven metabolic dysfunction. Our study demonstrated that NPC86 significantly downregulated inflammatory gene expression, including IL-1β and other cytokine-mediated immune activation pathways. A Western blot analysis confirmed a significant decrease in IL-1β levels in adipose tissue, validating the transcriptomic findings and reinforcing role of NPC86 in attenuating metabolic inflammation. In addition to its local anti-inflammatory effects in adipose tissue, NPC86 also significantly lowered the circulating levels of IL-1β and GM-CSF, suggesting a systemic immunomodulatory effect. Elevated IL-1β is a hallmark of metabolic inflammation and contributes to both impaired insulin secretion and β-cell apoptosis, thereby accelerating the progression of type 2 diabetes [43,44]. Similarly, increased circulating GM-CSF has been positively correlated with glycated hemoglobin in individuals with T2D, highlighting its association with poor glycemic control [45]. Previously, it was demonstrated that GAS5 sequesters the glucocorticoid receptor to regulate immune and inflammatory pathways, further supporting its role in modulating inflammatory responses [52].

This study provides strong evidence for the therapeutic potential of NPC86 in improving insulin sensitivity and metabolic function in DIOD mice. While these findings are promising, there are aspects that warrant further investigation. One consideration is that this study was conducted in male mice. Given the well-documented sex-based differences in metabolic regulation and insulin signaling, future studies incorporating both male and female cohorts will be essential to fully understand the potential of NPC86 across sexes and are currently being pursued by our lab. Additionally, an ongoing project compares NPC86 with other T2D medications.

Additionally, the treatment duration was limited to five days, which provides valuable insights into its safety and efficacy in vivo but does not capture long-term metabolic adaptations. Extending the treatment period in future studies will be important to evaluate the sustained impact of NPC86 on glucose metabolism, insulin sensitivity, and any potential long-term effects.

Finally, the systemic administration of NPC86, while effective, does not allow for precise organ-specific targeting. Exploring targeted delivery strategies, such as nanoparticle encapsulation, could enhance therapeutic efficacy by directing NPC86 to specific metabolic tissues, such as adipose tissue, the liver, or the pancreas. To address this, we have initiated studies investigating nanoparticle-based delivery methods to refine NPC86’s targeted therapeutic applications.

Overall, these results underscore the regulatory effects of NPC86 on insulin signaling, mitochondrial function, redox balance, lipid metabolism, and inflammatory pathways, highlighting its potential to modulate metabolic balance in insulin-resistant states. The integration of pathway analysis, clustering, and gene expression correlation supports NPC86 as a promising therapeutic candidate for metabolic disease intervention. Further pharmacokinetic and pharmacodynamic (PK/PD) studies of NPC86 are currently ongoing to evaluate its biodistribution, bioavailability, and long-term therapeutic potential.

## 4. Materials and Methods

### 4.1. Animal Study

All animal procedures were approved by the James A. Haley Veteran’s Hospital and University of South Florida Institutional Animal Care and Use Committee (IACUC) and conducted in compliance with the Animal Welfare Act (AWA), Health Research Extension Act (HREA), and ARRIVE guidelines. The mice were housed in a pathogen-free environment in plastic, sawdust-covered cages under a normal light–dark cycle with ad libitum access to food and water. Ten-week-old male, normal lean non-diabetic (ND) and diet-induced obese diabetic (DIOD) C57BL/6J mice were obtained from Jackson Laboratory. The mice were acclimated for two weeks, after which the DIOD mice were continued on a high-fat diet (60 kcal% fat, Research Diets; Catalog #D12492i), while the ND control mice were fed a regular diet (10 kcal% fat, Research Diets; Catalog #D12450Bi). NPC86 was administered via intraperitoneal (IP) injection starting at 12 weeks of age and continued daily for five consecutive days. The mice were euthanized at 13 weeks of age using CO_2_ anesthesia, followed by cervical dislocation as a secondary method of mortality confirmation. Tissue samples, including liver, spleen, kidney, heart, and white adipose tissue from anterior and posterior subcutaneous and visceral depots, were harvested, pooled, and analyzed from the ND, DIOD, and DIOD + NPC86-treated mice.

### 4.2. Hematoxylin and Eosin Staining

Following perfusion, liver, spleen, kidney, heart, and adipose tissues were collected and placed in 4% PFA solution for 24 h that was changed to 30% sucrose until completely sunk. The organs were sectioned into 30–35 μm slices using cryostat. These sections were placed on gelatin-coated slides and stored at 4 °C until staining. The following day, hematoxylin and eosin (H&E) staining was performed using the Abcam H&E kit (Abcam, Waltham, MA, USA; Catalog #ab245880), following the manufacturer’s protocol. The staining time varied between 30 s and 2 min depending on the tissue type. After staining, the slides were rinsed with distilled water to remove excess stain and then sequentially dehydrated using ethanol solutions of increasing concentrations (70%, 95%, and 100%, each for 2 min), followed by a final 2-min xylene wash. Cover slips were affixed using Permount mounting medium (Fisher Chemical, Pittsburg, PA, USA; Catalog #SP15-500). Tissue images were captured using the Keyence BZ-X810 digital integrated microscope.

### 4.3. Quantitative Real-Time PCR (RT-qPCR)

Total RNA was isolated from the adipose, heart, liver, spleen, and kidney tissues obtained from diet-induced obese diabetic (DIOD) and DIOD + NPC86-treated mice using RNAzol RT (Molecular Research Center Inc., Cincinnati, OH, USA; Catalog #RN190) according to the manufacturer’s instructions. RNA quality was assessed using 260/230 and 260/280 absorbance ratios to ensure purity. A total of 1 μg of RNA was reverse-transcribed into complementary DNA (cDNA) using the iScript™ cDNA Synthesis Kit (Bio-Rad, Hercules, CA, USA; Catalog #1708891). RT-qPCR was performed using a Power SYBR Green PCR Master Mix (Thermo Fisher Scientific, Waltham, MA, USA; Catalog #4367659) and run on an ABI ViiA7 sequence detection system (Applied Biosystems, Foster City, CA, USA). RT-qPCR reactions were performed in triplicate, including no-template and no-reverse transcriptase controls. Primer concentrations were optimized for a single melt curve and consistent amplification. The following primer sequences were used for amplification: β-actin (F: 5′-TGTCCACCTTCCAGCAGATGT-3′, R: 5′-AGCTCAGTAACAGTCCGCCTAGA-3′), gas5 exon12 (F: 5′-CTCCTGTGACAAGTGGAC-3′,R:5′-AACACAATATATCTGACACCATC-3′), and insulin receptor (F: 5′-AGATGAGAGGTGCAGTGTGGCT-3′, R: 5′-GGTTCCTTTGGCTCTTGCCACA-3′). Relative transcript expression levels were determined using the ∆∆CT method, normalizing target gene expression to β-actin. A standard curve was generated for each primer set to determine absolute quantification in ng (AQ), using cDNA from an ND mouse adipose tissue sample. The concentration of the amplified PCR product was measured using a DeNovix DS-11 spectrophotometer, and a serial dilution was performed (1:10, 1:100, 1:1000, 1:10,000, and 1:100,000) for a standard curve analysis. RT-qPCR was conducted using the standard curve protocol on the ABI ViiA7 system, and after the run, the expression levels were exported to Excel for AQ equation and standard curve generation.

### 4.4. RNAseq and Ingenuity Path Analysis

RNA was isolated from the adipose tissue of the ND, DIOD, and DIOD + NPC86 mice (500 ng/kg b.w. and 1 μg/kg b.w., *n* = 4 per group). To maximize biological diversity, RNA from three mice per group was pooled and sent for mRNA sequencing. RNA concentration was measured, and quality was verified using Qubit (Thermo Fisher, Waltham, MA, USA) and Agilent TapeStation (Agilent, Santa Clara, CA, USA), ensuring RIN > 8.0. Library preparation was performed using the TruSeq Stranded mRNA Library Prep Kit (Illumina, San Diego, CA, USA, Cat#: 20040532) according to the manufacturer’s instructions. The final DNA library concentration and quality were confirmed again using Qubit and TapeStation. Sequencing was performed in-house at the USF Genomics Sequencing Core using the Illumina NextSeq 2000 system with the 200-cycle (2 × 100 bp) P2 Reagent Kit. Each run yields approximately 800 million paired-end reads, with about 80 million paired-end reads generated per sample, with over 80% alignment to the reference mouse genome GRCm38. Reads were trimmed with Trimmomatic and quality-checked using FASTQC (v 0.12.1). FASTQ files were split to accelerate processing. Reads were mapped to the GRCm38 reference genome (downloaded from NCBI) using HISAT2 (v 2.1.0), followed by conversion with SAMtools (v 1.3.1). FeatureCounts was used to assign the mapped reads to genomic features. A differential gene expression analysis was performed using the Limma package in R (v3.62.2). *p*-values were calculated using a moderated t-statistic, and Benjamini–Hochberg (BH) correction was applied to adjust for multiple comparisons. For pathway and functional enrichment analysis, both KEGG and Gene Ontology (GO) term analyses were conducted. Additionally, the results were further explored using Qiagen’s Ingenuity Pathway Analysis (IPA) platform.

### 4.5. Automated Western Blot Analysis

An automated Western Blot analysis using the JESS system (ProteinSimple, Santa Clara, CA, USA) was performed on adipose tissue lysates following the manufacturer’s instructions. The amount of lysate to antibody was optimized, and a concentration of 1 mg/mL was used for all antibodies. The samples were separated on 12–230 kDa Wes Separation Module capillary cartridges of the Simple Protein Wes system. The following primary antibodies were used: IL-1β (R&D Systems, Minneapolis, MN, USA; Cat# AF-401-NA), insulin receptor (IR) (Oncogene, Oslo, Norway; Cat# GR07-100ug), total AKT 1/2/3 (Cell Signaling Technology, Danvers, MA, USA; Cat# 9272), phosphorylated AKT (p-AKT S473) (Cell Signaling Technology, Danvers, MA, USA; Cat# 9271), β-actin (Cell Signaling Technology, Danvers, MA, USA; Cat# 4970), and GAPDH (GenScript, Piscataway, NJ, USA; Cat#A00191). Each antibody was used at a dilution of 1:10. β-actin was used as a loading control. Secondary antibody module kits, specific for Jess (ProteinSimple, Santa Clara, CA, USA), were used, including Luminol-S, Peroxide, Streptavidin-HRP, anti-rabbit, and anti-mouse secondary antibody. Proteins were separated using capillary electrophoresis. Compass software (ProteinSimple v 6.3.0) was used to analyze the chemiluminescence signal peaks that were generated. Digital images corresponding to traditional Western blot bands were generated by Compass software.

### 4.6. ELISA

The quantification of inflammatory cytokines was performed using the Mouse Inflammation ELISA Strip Kit (Signosis, Santa Clara, CA, USA; Catalog #EA-1051), following the manufacturer’s protocol. The colorimetric sandwich-based assay was used to detect IL-1β and GM-CSF in the mouse serum. Individual wells of the 96-well plate were pre-coated with specific capture antibodies against these cytokines. Serum samples were collected from the DIOD mice and DIOD + NPC86-treated mice (500 ng/kg and 1 µg/kg body weight). The samples were diluted 1:10 in 1× diluent buffer, and 100 μL was added per well. The plates were incubated for 2 h at room temperature with gentle agitation. Following three washes with 1× assay wash buffer, the wells were incubated with 100 μL of a 1:50 diluted biotin-conjugated detection antibody mixture for 1 h. The plates were washed again three times and then incubated with 100 μL of 1:200 diluted streptavidin–HRP conjugate for 45 min at room temperature with gentle shaking. A final wash step was performed before adding 100 μL of the TMB substrate to each well for 30 min. The reaction was stopped by adding 50 μL of a stop solution, and absorbance was measured at 450 nm using a BioTek Synergy MX microplate reader (BioTek Instruments, Inc. (Agilent Technologies), Winooski, VT, USA). Data acquisition and analysis were performed using BioTek Gen5 software (Gen5 1.10). The absolute quantification of cytokine levels was achieved using the Mouse Inflammation ELISA Strip Protein Standards Kit (Signosis, Catalog #EA-1052). Standard curves were generated by 2-fold serial dilution (8 ng/mL to 0.5 ng/mL) of the protein standards for IL-1β and GM-CSF in 1× diluent buffer. All data are presented as mean ± SEM. Statistical comparisons between groups were performed using an unpaired *t*-test in GraphPad Prism v10.0.0. Significance thresholds were set at * *p* < 0.05 and ** *p* < 0.01.

### 4.7. Statistical Analysis

All experiments were conducted in triplicate to ensure the reproducibility of results. The data analysis was performed using GraphPad PRISM™ software (version 10.0.0). To determine statistical significance between the groups, either Student’s *t*-test or one-way ANOVA was applied, depending on the variance equality. Statistical significance was defined as *p* < 0.05 (*), *p* < 0.01 (**), *p* < 0.001 (***), and **** *p* < 0.0001, while *p* ≥ 0.05 was considered not significant (ns).

## 5. Conclusions

**Figure 11:**
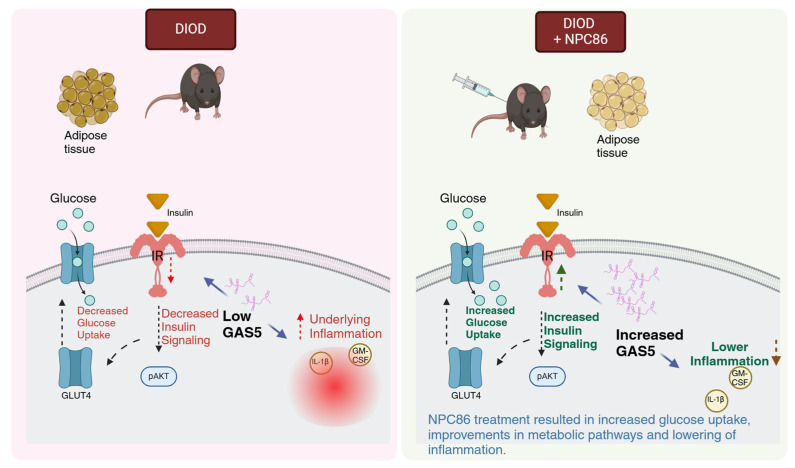
Schematic illustrating how increased GAS5 levels enhance insulin signaling and glucose uptake while concurrently reducing inflammatory responses, created in BioRender. Patel, N. (2025). Reprinted with permission from Patel, N (2025). Copyright 2025, Patel, N.

T2D is a progressive metabolic disorder driven by insulin resistance and impaired glucose metabolism. This study demonstrates (schematic in Figure 11) that NPC86, a small-molecule therapeutic compound, increases gas5 levels, enhances insulin signaling and metabolic function in DIOD mice, highlighting the therapeutic potential of lncRNA-targeting strategies in T2D. NPC86 treatment increased gas5 expression, leading to higher IR levels, enhanced AKT phosphorylation, and improved glucose clearance. The RNAseq analysis revealed that NPC86 modulates oxidative phosphorylation and glycolysis while suppressing inflammation, suggesting a multi-pathway mechanism for improving insulin sensitivity. Since mitochondrial dysfunction and chronic inflammation contribute to insulin resistance, these findings support NPC86 as a promising candidate for metabolic intervention in T2D. This study shows the efficacy and safety of a lncRNA-targeting small molecule in a diabetes model in vivo. The results thus provide a broader understanding of the therapeutic potential of small molecules targeting lncRNAs in other diseases.

## 6. Patents

1. US Patent No. 10,724,097 Methods and compositions for diagnosis and management of diabetes and metabolic syndrome (28 July 2020)—NAP.
2. US Patent No. 11,214,835 Methods and compositions for diagnosis and management of neurodegenerative diseases (4 January 2022)—NAP, JC.
3. US Patent No. 11,278,521 GAS5 binding compounds, formulations and uses thereof (22 March 2022)—NAP, JC.

## Data Availability

The original RNASeq dataset presented in this study are included in the Supplementary Materials. Future inquiries can be directed to the corresponding author.

## Supplementary Materials

The following supporting information can be downloaded at https://www.mdpi.com/article/10.3390/ijms26083695/s1.

## References

[1] Stratton I.M., Adler A.I., Neil H.A.W., Matthews D.R., Manley S.E., Cull C.A., Hadden D., Turner R.C., Holman R.R. (2000). Association of glycaemia with macrovascular and microvascular complications of type 2 diabetes (UKPDS 35): Prospective observational study. BMJ.

[2] CDC (2024). A Report Card: Diabetes in the United States Infographic, Diabetes. https://www.cdc.gov/diabetes/communication-resources/diabetes-statistics.html.

[3] Centers for Disease Control and Prevention (2023). National Diabetes Statistics Report, 2023: Estimates of Diabetes and Its Burden in the United States.

[4] Rowley W.R., Bezold C., Arikan Y., Byrne E., Krohe S. (2017). Diabetes 2030: Insights from Yesterday, Today, and Future Trends. Popul. Health Manag..

[5] Chamine I., Hwang J., Valenzuela S., Marino M., Larson A.E., Georgescu J., Latkovic-Taber M., Angier H., DeVoe J.E., Huguet N. (2022). Acute and Chronic Diabetes-Related Complications Among Patients With Diabetes Receiving Care in Community Health Centers. Diabetes Care.

[6] Eibner C., Krull H., Brown K.M., Cefalu M., Mulcahy A.W., Pollard M., Shetty K., Adamson D.M., Amaral E.F.L., Armour P. (2016). Current and Projected Characteristics and Unique Health Care Needs of the Patient Population Served by the Department of Veterans Affairs. Rand Health Q..

[7] Narayan K.M.V., Boyle J.P., Thompson T.J., Gregg E.W., Williamson D.F. (2007). Effect of BMI on lifetime risk for diabetes in the U.S. Diabetes Care.

[8] Yu C., Benhammou J.N., Goyal D., Oh D., Wang L., Jacobs J., Dixit V., Tache Y., Pisegna J. (2016). High Protein Dietary Intervention Improves Body Mass Index (BMI) and Reduces the NAFLD Fibrosis Score (NFS) in Veterans with Obesity: 783. Off. J. Am. Coll. Gastroenterol. ACG.

[9] Boyko E.J., Jacobson I.G., Smith B., Ryan M.A.K., Hooper T.I., Amoroso P.J., Gackstetter G.D., Barrett-Connor E., Smith T.C. (2010). Risk of Diabetes in U.S. Military Service Members in Relation to Combat Deployment and Mental Health. Diabetes Care.

[10] Batista P.J., Chang H.Y. (2013). Long Noncoding RNAs: Cellular Address Codes in Development and Disease. Cell.

[11] Taniue K., Akimitsu N. (2021). The Functions and Unique Features of LncRNAs in Cancer Development and Tumorigenesis. Int. J. Mol. Sci..

[12] Zhu M., Chen Q., Liu X., Sun Q., Zhao X., Deng R., Wang Y., Huang J., Xu M., Yan J. (2014). lncRNA H19/miR-675 axis represses prostate cancer metastasis by targeting TGFBI. FEBS J..

[13] Zhao Q., Li T., Qi J., Liu J., Qin C. (2014). The miR-545/374a cluster encoded in the Ftx lncRNA is overexpressed in HBV-related hepatocellular carcinoma and promotes tumorigenesis and tumor progression. PLoS ONE.

[14] Faghihi M.A., Modarresi F., Khalil A.M., Wood D.E., Sahagan B.G., Morgan T.E., Finch C.E., Laurent G.S., Kenny P.J., Wahlestedt C. (2008). Expression of a noncoding RNA is elevated in Alzheimer’s disease and drives rapid feed-forward regulation of β-secretase expression. Nat. Med..

[15] Amaral P.P., Clark M.B., Gascoigne D.K., Dinger M.E., Mattick J.S. (2011). lncRNAdb: A reference database for long noncoding RNAs. Nucleic Acids Res..

[16] International Human Genome Sequencing Consortium (2001). Initial sequencing and analysis of the human genome. Nature.

[17] Smith C.M., Steitz J.A. (1998). Classification of gas5 as a Multi-Small-Nucleolar-RNA (snoRNA) Host Gene and a Member of the 5′-Terminal Oligopyrimidine Gene Family Reveals Common Features of snoRNA Host Genes. Mol. Cell. Biol..

[18] Mazar J., Rosado A., Shelley J., Marchica J., Westmoreland T.J. (2016). The long non-coding RNA GAS5 differentially regulates cell cycle arrest and apoptosis through activation of BRCA1 and p53 in human neuroblastoma. Oncotarget.

[19] Tani H., Torimura M., Akimitsu N. (2013). The RNA degradation pathway regulates the function of GAS5 a non-coding RNA in mammalian cells. PLoS ONE.

[20] Raho G., Barone V., Rossi D., Philipson L., Sorrentino V. (2000). The gas 5 gene shows four alternative splicing patterns without coding for a protein. Gene.

[21] Carter G., Miladinovic B., Patel A.A., Deland L., Mastorides S., Patel N.A. (2015). Circulating long noncoding RNA GAS5 levels are correlated to prevalence of type 2 diabetes mellitus. BBA Clin..

[22] Shi Y., Parag S., Patel R., Lui A., Murr M., Cai J., Patel N.A. (2019). Stabilization of lncRNA GAS5 by a Small Molecule and Its Implications in Diabetic Adipocytes. Cell Chem. Biol..

[23] Wang Y., Xue M., Xia F., Zhu L., Jia D., Gao Y., Li L., Shi Y., Li Y., Chen S. (2022). Long Non-Coding RNA GAS5 in Age-Related Diseases. Curr. Med. Chem..

[24] Luo Y., Guo J., Xu P., Gui R. (2020). Long Non-coding RNA GAS5 Maintains Insulin Secretion by Regulating Multiple miRNAs in INS-1 832/13 Cells. Front. Mol. Biosci..

[25] Zhang L., Zhao S., Zhu Y. (2020). Long noncoding RNA growth arrest-specific transcript 5 alleviates renal fibrosis in diabetic nephropathy by downregulating matrix metalloproteinase 9 through recruitment of enhancer of zeste homolog 2. FASEB J. Off. Publ. Fed. Am. Soc. Exp. Biol..

[26] Sun H., Chen T., Li X., Zhu Y., Zhang S., He P., Peng Y., Fan Q. (2023). The relevance of the non-invasive biomarkers lncRNA GAS5/miR-21 ceRNA regulatory network in the early identification of diabetes and diabetic nephropathy. Diabetol. Metab. Syndr..

[27] Degerman E., Ahmad F., Chung Y.W., Guirguis E., Omar B., Stenson L., Manganiello V. (2011). From PDE3B to the regulation of energy homeostasis. Curr. Opin. Pharmacol..

[28] Silver I.A., Erecińska M. (1994). Extracellular glucose concentration in mammalian brain: Continuous monitoring of changes during increased neuronal activity and upon limitation in oxygen supply in normo-, hypo-, and hyperglycemic animals. J. Neurosci..

[29] Huang S., Czech M.P. (2007). The GLUT4 glucose transporter. Cell Metab..

[30] Takino J., Sato T., Nagamine K., Hori T. (2019). The inhibition of Bax activation-induced apoptosis by RasGRP2 via R-Ras-PI3K-Akt signaling pathway in the endothelial cells. Sci. Rep..

[31] Oram J.F., Lawn R.M. (2001). ABCA1: The gatekeeper for eliminating excess tissue cholesterol. J. Lipid Res..

[32] Olivecrona G. (2016). Role of lipoprotein lipase in lipid metabolism. Curr. Opin. Lipidol..

[33] Wang Q.A., Zhang F., Jiang L., Ye R., An Y., Shao M., Tao C., Gupta R.K., Scherer P.E. (2018). Peroxisome Proliferator-Activated Receptor γ and Its Role in Adipocyte Homeostasis and Thiazolidinedione-Mediated Insulin Sensitization. Mol. Cell. Biol..

[34] Moreno-Fernandez M.E., Giles D.A., Stankiewicz T.E., Sheridan R., Karns R., Cappelletti M., Lampe K., Mukherjee R., Sina C., Sallese A. (2018). Peroxisomal β-oxidation regulates whole body metabolism, inflammatory vigor, and pathogenesis of nonalcoholic fatty liver disease. JCI Insight.

[35] Lubos E., Loscalzo J., Handy D.E. (2011). Glutathione peroxidase-1 in health and disease: From molecular mechanisms to therapeutic opportunities. Antioxid. Redox Signal..

[36] Ohmori I., Ouchida M., Imai H., Ishida S., Toyokuni S., Mashimo T. (2022). Thioredoxin deficiency increases oxidative stress and causes bilateral symmetrical degeneration in rat midbrain. Neurobiol. Dis..

[37] Klopotowska M., Bajor M., Graczyk-Jarzynka A., Kraft A., Pilch Z., Zhylko A., Firczuk M., Baranowska I., Lazniewski M., Plewczynski D. (2022). PRDX-1 supports the survival and antitumor activity of primary and CAR-modified NK cells under oxidative stress. Cancer Immunol. Res..

[38] Hao Y.Y., Xiao W.Q., Zhang H.N., Yu N.N., Park G., Han Y.H., Kwon T., Sun H.N. (2024). Peroxiredoxin 1 modulates oxidative stress resistance and cell apoptosis through stemness in liver cancer under non-thermal plasma treatment. Biochem. Biophys. Res. Commun..

[39] Miao R., Fang X., Wei J., Wu H., Wang X., Tian J. (2022). Akt: A Potential Drug Target for Metabolic Syndrome. Front. Physiol..

[40] Jurca C.M., Kozma K., Petchesi C.D., Zaha D.C., Magyar I., Munteanu M., Faur L., Jurca A., Bembea D., Severin E. (2023). Tuberous Sclerosis, Type II Diabetes Mellitus and the PI3K/AKT/mTOR Signaling Pathways—Case Report and Literature Review. Genes.

[41] Wang Q., Li M., Zeng N., Zhou Y., Yan J. (2023). Succinate dehydrogenase complex subunit C: Role in cellular physiology and disease. Exp. Biol. Med..

[42] Jones J.M., Datta P., Srinivasula S.M., Ji W., Gupta S., Zhang Z., Davies E., Hajnóczky G., Saunders T.L., Van Keuren M.L. (2003). Loss of Omi mitochondrial protease activity causes the neuromuscular disorder of mnd2 mutant mice. Nature.

[43] Tong H.V., Luu N.K., Son H.A., Hoan N.V., Hung T.T., Velavan T.P., Toan N.L. (2017). Adiponectin and pro-inflammatory cytokines are modulated in Vietnamese patients with type 2 diabetes mellitus. J. Diabetes Investig..

[44] Sepehri Z., Kiani Z., Afshari M., Kohan F., Dalvand A., Ghavami S. (2017). Inflammasomes and type 2 diabetes: An updated systematic review. Immunol. Lett..

[45] Surendar J., Mohan V., Pavankumar N., Babu S., Aravindhan V. (2012). Increased levels of serum granulocyte-macrophage colony-stimulating factor is associated with activated peripheral dendritic cells in type 2 diabetes subjects (CURES-99). Diabetes Technol. Ther..

[46] Alharbi K.S. (2024). GAS5: A pivotal lncRNA in diabetes mellitus pathogenesis and management. Pathol.-Res. Pract..

[47] Boucher J., Kleinridders A., Kahn C.R. (2014). Insulin Receptor Signaling in Normal and Insulin-Resistant States. Cold Spring Harb. Perspect. Biol..

[48] Tonks K.T., Ng Y., Miller S., Coster C.A., Samocha-Bonet G.K., Iseli L.V., Xu J.S., Ye L., Allen D.E., Desilva H. (2013). Impaired Akt phosphorylation in insulin-resistant human muscle is accompanied by selective and heterogeneous downstream defects. Diabetologia.

[49] Sang L., Ju H.Q., Yang Z., Ge Q., Zhang Z., Liu F., Yang L., Gong H., Shi C., Qu L. (2021). Mitochondrial long non-coding RNA GAS5 tunes TCA metabolism in response to nutrient stress. Nat. Metab..

[50] Rovira-Llopis S., Bañuls C., Diaz-Morales N., Hernandez-Mijares A., Rocha M., Victor V.M. (2017). Mitochondrial dynamics in type 2 diabetes: Pathophysiological implications. Redox Biol..

[51] Hurrle S., Hsu W.H. (2017). The etiology of oxidative stress in insulin resistance. Biomed. J..

[52] Kino T., Hurt D.E., Ichijo T., Nader N., Chrousos G.P. (2010). Noncoding RNA gas5 is a growth arrest- and starvation-associated repressor of the glucocorticoid receptor. Sci. Signal..

