# Supplementary figures and images for: NPC86 Increases LncRNA Gas5 In Vivo to Improve Insulin Sensitivity and Metabolic Function in Diet-Induced Obese Diabetic Mouse Model
Source: Int J Mol Sci. 2025 Apr 14;26(8):3695. doi: 10.3390/ijms26083695 (PMC12027414; doi:10.3390/ijms26083695)

IL-1 $\beta$

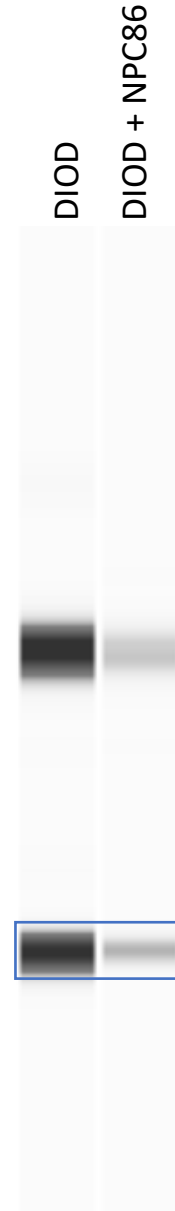

GAPDH

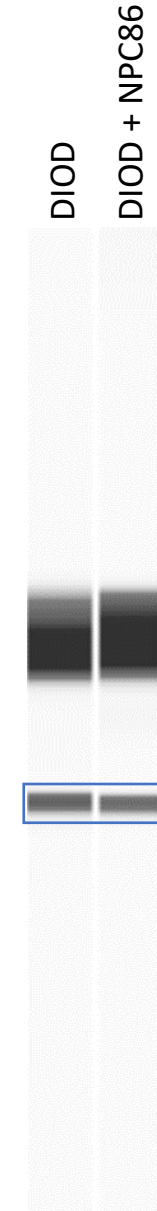

p-AKT

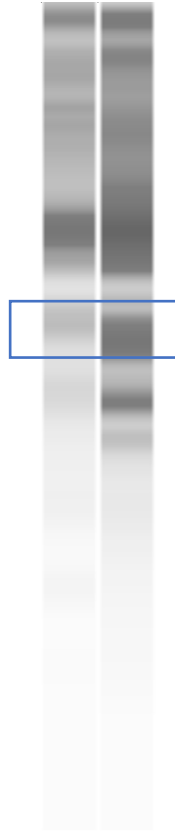

AKT

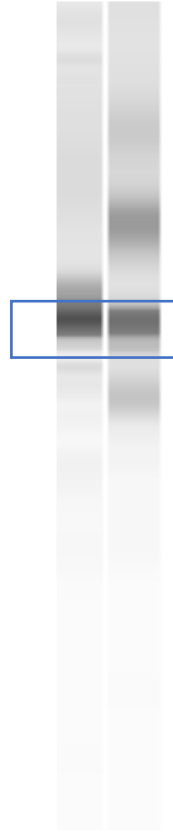

B-actin

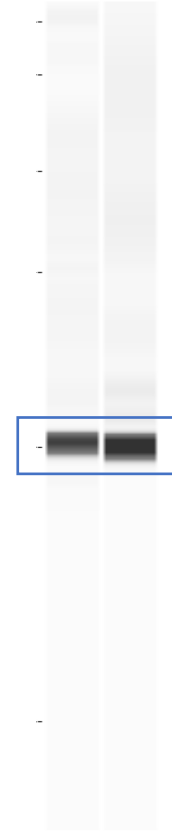

IR

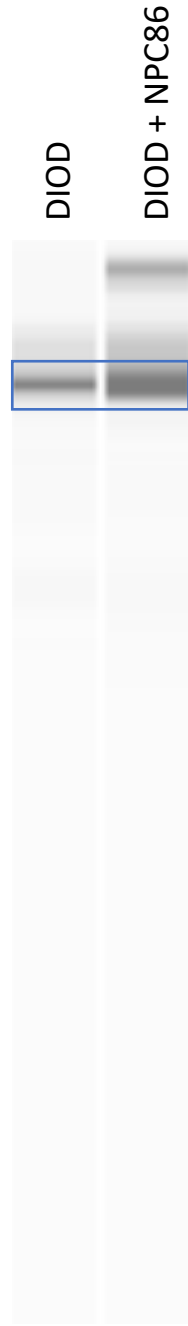

$\beta$ -Actin

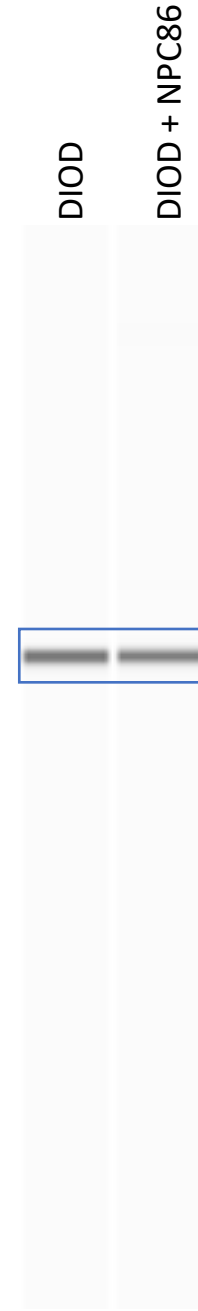

Supplement: Supplementary file 1 [file ijms-26-03695-s001.zip › IJMS Original Blots.pdf]
